# Fostering cultural inclusiveness and learning in culturally mixed business classes

**DOI:** 10.1186/2193-1801-3-242

**Published:** 2014-05-12

**Authors:** Anita S Mak, Anne Daly, Michelle C Barker

**Affiliations:** Faculty of Health, University of Canberra, Canberra, ACT 2601 Australia; Faculty of Business, Government and Law, University of Canberra, Canberra, ACT 2601 Australia; Department of International Business and Asian Studies, Griffith University, Nathan Campus, Brisbane, QLD 4111 Australia

## Abstract

**Background:**

Business educators have advocated that in order to build faculty’s intercultural capability, it is vital to provide them with professional development in using intercultural training resources and with “community of practice” support in adapting such resources for enhancing their students’ intercultural learning. This approach has been adopted in an Australian action research project titled “Internationalisation at Home” (IaH), which involved providing faculty with professional development adapted from an established intercultural training resource - the EXCELL (Excellence in Cultural Experiential Learning and Leadership) Program.

**Case description:**

In this paper, we present two case studies of the implementation of the IaH Project in business schools at the University of Canberra and at Griffith University. Lessons learned from the first study were incorporated in the design and evaluation of the second one. Faculty leaders will describe how they engage and support colleagues in adapting components of EXCELL to foster cultural inclusiveness and facilitate students’ intercultural competence development. As part of project evaluation, we hypothesised that students who participated in IaH courses would report greater levels of (1) cultural inclusiveness in their educational environment, and (2) cultural learning development, compared with students who were not enrolled in IaH courses. Research participants in the Canberra case study comprised an intervention group of 140 business undergraduates enrolled in an IaH course, and a control group of 59 non-IaH undergraduates. At Griffith, participants were 211 first year management students in the intervention group and 84 students enrolled in a non-IaH first year course.

**Discussion and evaluation:**

In each case study, an end-of-semester survey showed that students who had completed courses with the IaH project intervention reported significantly greater levels of perceived cultural inclusiveness in multicultural classes, and of cultural learning development, than students in the control group. Faculty’s reflections on project processes and outcomes further suggest that implementing strategic, structured active learning interventions such as in the IaH Project, could bring about more productive social interactions in multicultural classes and benefit domestic and especially international students. We will discuss implications of the findings for students’ intercultural learning, faculty’s needs for continual professional development, and the role of institutional support in intercultural competence development.

## Background

Increasing cultural diversity in the student body has become characteristic of business higher education. The number of foreign tertiary education students enrolled outside of their country of origin steadily increased by 7.1% per year in the 11-year period from 2000, to 4.1 million such students worldwide in 2010 (OECD 2012). While the top three destination countries (the United States, the United Kingdom, and Australia) were English-speaking countries, the top three sources were Asian countries (China, India, and the Republic of Korea). Among the OECD countries, Australia hosted the largest proportion (at over 21%) of international students among onshore tertiary students, with business studies being the most popular field of studies.

The increased cultural diversity in classes and on campus exposes domestic students to international students from culturally diverse backgrounds; such contact can potentially enhance intergroup relations across racial and cultural boundaries (Pettigrew and Tropp 2006). Domestic business students can also gain international perspectives and increase their intercultural knowledge and skills through interacting with international students without leaving their home university. The term “internationalisation at home” (Crowther et al. 2003) was coined to describe this process. More recently, internationalisation agendas have become prominent in universities’ strategic plans (Elkin et al. 2008; Mak 2010) and are reflected in graduate attributes linked to global citizenship (Lilley et al. 2014). In order for all students to achieve such desirable attributes as global citizenship, educators need to develop effective strategies to foster cultural inclusiveness and effective intercultural interactions in classrooms and on campuses (Barker and Mak 2013).

Notably, research has shown generally low levels of intercultural contact and friendships between Australian-born students and international students from diverse cultural backgrounds (Mak et al. 2014; Summers and Volet 2008; Volet and Ang 2012). Being in a culturally diverse educational environment, such as in Australian higher education, does not automatically induce improvements in the quality of interactions between domestic and international students from diverse linguistic backgrounds, or increases in intercultural knowledge and skills of domestic students (Arkoudis et al. 2010; Leask 2009). Australian teachers have also observed that multicultural group work for business students, while beneficial for preparing globally aware and work ready graduates, to be particularly challenging (Woods et al. 2011). International students describe feeling devalued by domestic students in assessment groups, and frequently withdraw from participation because of lack of confidence in speaking English and understanding colloquial expressions. Domestic students often express frustration that international students only want to work with peers from their country of origin and that their contribution may detract from the quality of work produced by the group. Interestingly, there is evidence that social interactions with domestic students could facilitate international students’ psychological adaptation in the host country, an important outcome for international education (Ward and Masgoret 2004; Zhang and Goodson 2011).

In a critical review of contemporary practice and research in internationalisation within business education, Caruana and Ploner (2012) note the challenges of culturally mixed group work. They also highlight the utility of developing and extending faculty skills in managing diversity and of experiential learning for internationalisation of student outlooks. In another critical review of contemporary practice that focuses on training in intercultural competence in business education, Hall et al. (2012) argue that intercultural competence development in students can best be viewed as a process of “intercultural learning” that involves teachers (in their role as intercultural educators) understanding themselves to be also intercultural learners and who will benefit from “the training of trainers” (see also Cushner and Mahon 2009; Paige and Goode 2009). Hall and colleagues emphasise teachers’ “important role in intervening to facilitate intercultural interaction, and to equip students with conceptual models and frameworks for reflective learning” (p. 13).

Other educators (e.g., Freeman et al. 2009; Sanderson 2008) have argued that cognitive understanding of intercultural competence alone is insufficient to bring about deep intercultural learning in either teachers or their students. Moreover, Freeman and colleagues have identified a lack of literature on the “how to” in embedding intercultural competence development in business education. Consistent with the training-the-trainer approach (Hall et al. 2012), however, scholars (Caruana and Ploner 2012; Freeman et al. 2009; Ward 2006) recommend the use of existing evidence-based intercultural training resources, such as the EXCELL (**Ex**cellence in **C**ultural **E**xperiential **L**earning and **L**eadership) Program, to upskill teachers to be effective intercultural educators. EXCELL focuses on developing participants’ generic social competencies in communicating and working with cultural others, in several key areas of interactions - seeking help, making social contact, participation in a group, refusing a request, expressing disagreement and giving feedback. The complete training model involves building awareness and respect about participants’ cultural backgrounds (the Alliance Building tool), the development of a schematic representation of verbal and non-verbal behaviours along with the cultural values underpinning the behaviours (the Cultural Mapping tool), demonstrations by facilitators of each competency, practice by participants, and coaching and feedback from facilitators about participants’ skills (Mak et al. 1998). The incorporation of the full EXCELL Program in the curriculum, which generally requires six group sessions of two to three hours, was found to be effective in increasing students’ social interaction skills, intercultural social self-efficacy, and time spent with people from other ethnic groups (Ho et al. 2004; Mak et al. 1999a; Mak and Buckingham 2007). For further information about the EXCELL Program, refer to Mak et al. (1999b) for its conceptual framework, and to Westwood et al. (2000) for its teaching processes. Mak (2011) outlines applications of EXCELL for facilitating international students’ intercultural interactions.

Freeman et al. (2009) have advocated that program leaders should engage communities of academics such as within the one discipline or school (as a “community of practice” approach) in embedding inclusive teaching practices and intercultural competence development in the formal curriculum, and evaluate the subsequent impact on student outcomes. Intercultural competence development can best be understood as “a dynamic, ongoing, interactive, self-reflective learning process that transforms attitudes, skills and knowledge for effective communication and interaction across a range of cultures and contexts” (Freeman et al. 2009, p.13). The approach recommended by Freeman and colleagues has been adopted in a recently completed Australian project titled “Internationalisation at Home (IaH): Enhancing Intercultural Capabilities of Business and Health Teachers, Students, and Curricula”. The two-year IaH Project (2011–2012) was funded by the Australian Government Office for Learning and Teaching. It involved providing professional development programs based on two components of EXCELL, namely, Alliance Building and Cultural Mapping. The programs aimed to enhance academics’ skills to explicitly respect and utilise cultural differences among students as part of the curriculum. For more information on the IaH Project, visit the project website at https://sites.google.com/site/internationalisationathome.

Alliance Building activities aim to build safety in groups and encourage participation and sharing of experiences of observations (Mak et al. 1998). Cultural Mapping provides a schematic framework for breaking down social interactions in a given intercultural encounter into manageable phases (see https://sites.google.com/site/internationalisationathome/professional-development for examples of cultural maps relevant to multicultural business classes). A cultural map offers a structured and succinct description of one effective and culturally appropriate way of behaving – both verbal and nonverbal – in a given social scenario. In a pilot teaching project on which the IaH Project was based, Mak and Kennedy (2012) found that training faculty in the use of Alliance Building and Cultural Mapping could prepare and support them in embedding intercultural skills development in their teaching. For example, an accounting academic implemented an experiential Alliance Building activity called the Name Game. Pairs of students engaged initially in a structured sharing of information about each other’s names and cultures and later shared this discussion with the entire class. These activities taught students how to talk to peers from different cultural groups and how to validate each individual’s cultural origins.

In this paper, we present two case studies of the implementation of the IaH Project in business schools in Canberra and South East Queensland. The overall IaH Project was based on the action research cycle framework of planning, acting, observing, and reflecting. Collaborative action research involves participants sharing common interests, working together to innovate, reform the curriculum, and to evaluate and reflect on their processes (Haggarty and Postlethwaite 2003). Lessons learned from the trial implementation of the project at the Faculty of Business, Government, and Law at the University of Canberra, were incorporated in the design and evaluation of the trial at Griffith Business School at Griffith University in Queensland.

## The Internationalisation at Home (IaH) Project in business schools

Building on previous work by Mak and Kennedy (2012) and Freeman et al. (2009), the IaH Project aimed to internationalise the learning and teaching practices of business and health higher education, through intercultural capacity-building of teachers, international students, and domestic students. The initial phases of the project involved consulting stakeholder groups and conduct of subsequent professional development at two Australian business schools (see Mak et al. 2013). Analyses of focus groups with business professionals, faculty, domestic students, and international students, generated recurrent challenging intercultural social scenarios (or critical incident scenarios) in business classes and workplaces. These real-life scenarios were used in a professional development workshop on Building Intercultural Competencies for faculty at the business schools. Mak et al. (2013) report how the workshop incorporated the stakeholder-generated scenarios with the EXCELL Alliance Building and Cultural Mapping tools, and the positive end-of-workshop evaluation by participating business faculty.

Following the workshops, a faculty leader in each of the business schools set up a Learning Circle in Intercultural Skills, to support workshop participants and other colleagues to adapt the workshop training in the use of one or both of the EXCELL tools, for embedding intercultural competence development in their pedagogy and curriculum development. The Learning Circle provided a forum for academics to share the initiatives they were implementing, to draw on each other’s experience, and to reflect on the successes and challenges of their interventions.

In each of the case studies below, the Learning Circle leader will report its community of practice processes and outcomes in the relevant institutional contexts. The focus will be on curriculum change outcomes undertaken by Learning Circle members and particularly on the associated student outcomes.

We will report student outcomes in intercultural competence development using two indicators – students’ perceived cultural inclusiveness of their educational environment and their personal cultural learning development. Cultural inclusiveness is an educational environmental condition that recognises and values diversity, and enables the world views of all students to be expressed through teaching and learning (McLoughlin 2011), thus contributing to increasing acceptance and interactions among students from diverse cultures (Thompson and Byrnes 2011). Student perceptions of their cultural learning development from undertaking a component of a program of studies, provide an indicator of their personal intercultural competence attainment linked to the formal curriculum. The present report will include results from testing two hypotheses about the efficacy of the IaH Project in terms of students’ perceptions of cultural inclusiveness and their cultural learning development.

Then, in the Discussion section, we will draw on the findings from the case studies to evaluate the project outcomes and lessons for business higher education. We will discuss the implications of the findings for students’ intercultural learning, continual professional development for faculty, and institutional support needed for embedding intercultural competence development.

## Hypotheses and methodology for evaluating student outcomes in each case study

As part of the project evaluation on student outcomes, we hypothesised that students who participated in the IaH courses (that is, units or subjects that make up a program of study) would report stronger levels of cultural inclusiveness in their educational environment (H1), and greater personal cultural learning development (H2), compared with students who were not enrolled in IaH courses.

## Method and procedure

After obtaining approval from the Human Research Ethics Committees at the University of Canberra and at Griffith University, we recruited student volunteers in both IaH and non-IaH classes to participate in an anonymous cross-sectional end-of-semester survey on students’ educational and intercultural experiences that took under 10 minutes to complete. The survey comprised a demographic section (including sex, age, relevant course enrolment, and residential status), as well as measures of perceived cultural inclusiveness and cultural learning development.

A 7-item measure on students’ perceptions of cultural inclusiveness in multicultural classes was adapted from Ward and Masgoret 2004 (see Table 1 for the items). The survey also included a 12-item measure of cultural learning development (see Table 2), with three items adapted from Mak (2012), six items adapted from MacNab and Worthley (2012), and three additional items composed by the first author. Items on both measures were presented as five-point rating scales, ranging from 1 = Strongly Agree, to 5 = Strongly Disagree. In this project, both scales attained satisfactory internal consistency, with reliability coefficients of 0.81 and 0.95, respectively. Moreover, a factor analysis conducted with all 19 items (using the principal component extraction method with oblimin rotation) yielded two factors, with 7 items loaded on a single factor of cultural inclusiveness, and 12 items loaded on another factor of cultural learning development, suggesting construct validity.

**Table 1:** **Perceived cultural inclusiveness among undergraduates in intervention and control groups (in% strongly agreed or agreed)**

Statement on perceived cultural inclusiveness	Canberra		Griffith	
	Intervention	Control	Intervention	Control
1. My teachers encourage contact between students from different cultural backgrounds.	92.1	50.0	67.8	41.0
2. My teachers make special efforts to help international students.	66.4	51.7	56.4	37.3
3. Cultural differences are respected in my university.	82.1	70.7	84.4	54.9
4. My teachers understand the problems of international students.	68.6	59.6	65.7	43.4
5. In my classes, there are opportunities for students to learn about different cultures.	67.9	43.1	61.6	45.8
6. My classmates are accepting of cultural differences.	71.4	66.7	78.2	51.8
7. Students from different cultural groups work well with each other in my classes.	64.3	51.7	62.6	36.1

**Table 2:** **Cultural learning development among undergraduates in intervention and control groups (in% strongly agreed or agreed)**

Statement on cultural learning	Canberra		Griffith	
	Intervention	Control	Intervention	Control
1. I have developed a greater awareness of cultural diversity.	72.9	39.0	64.3	48.8
2. I have developed a better understanding of cross-cultural interpersonal skills.	69.3	44.1	65.7	59.5
3. I have gained awareness of the role of culture in my chosen field of study.	65.0	45.8	68.6	59.0
4. I am now more conscious of the cultural knowledge I use when interacting with people with different cultural backgrounds.	68.6	54.2	69.0	54.8
5. I am now more conscious of the cultural knowledge I apply to cross-cultural interactions.	66.4	42.4	64.4	53.0
6. I am better prepared to adjust my cultural knowledge as I interact with people from an unfamiliar culture.	63.6	50.8	68.6	57.1
7. I am now better equipped to enjoy living in cultures that are unfamiliar to me.	62.9	54.2	54.8	52.4
8. I am now more confident that I could socialise with locals in a culture that is unfamiliar.	65.0	53.4	56.0	51.2
9. I am now more certain that I could deal better with adjusting to a culture that is new to me.	70.7	45.8	59.5	51.8
9. I have become more confident with communicating with people from culturally different backgrounds.	68.6	52.5	61.4	52.4
10. I have become more ready to make social contact with culturally different others.	69.3	45.8	62.4	47.6
11. I have become more comfortable participating in multicultural groups.	67.9	52.5	6	47.6

Using purposive sampling methods, participants were recruited from university classes with the permission of relevant course convenors. Depending on convenors’ preferences, students accessed the anonymous questionnaire either via a web link to a Qualtrics survey, or were invited to complete hard copies of the survey distributed in class. We also provided the option of returning completed paper surveys to the principal researcher using pre-paid envelopes. Students participating in the survey could choose to go into a lucky draw for a $100 shopping voucher, via either a separate web page (on Qualtrics) or a separate page distributed with the paper surveys in order to preserve anonymity.

### Case1: Faculty of Business, Government, & Law, University of Canberra

In common with business programs around Australia, there have been substantial changes in the composition of the student body in business degrees at the Faculty of Business, Government, and Law (BGL) at the University of Canberra. At the undergraduate level, about 40 per cent of BGL students are international students and the vast majority are from non-English-speaking backgrounds, with China, India and other Asian countries being the major sources. The recognition of the challenges and opportunities arising in a multicultural classroom was an important reason for BGL agreeing to join the IaH project. While there had been previous workshops and discussion about internationalisation within BGL, there had been limited systematic efforts to promote intercultural understanding both within and outside the classroom. The IaH project offered an opportunity to develop ideas and practical solutions on this important topic within BGL.

### IaH processes at BGL in Canberra

While progress on the project within BGL was informed by what was going on elsewhere in the project through meetings, teleconferencing and access to resources on the web, the implementation of this action research project was determined by the interest and commitment of people within BGL. The successful professional development workshop described earlier was followed by an invitation to all business faculty, both permanent and casual, to join a Learning Circle in Intercultural Skills with a focus on internationalisation and developing a community of practice surrounding this important topic.

The Learning Circle met two to three times each semester over an 18 month period and had a total of about 20 participants though some were more regular attendees than others. Participants at each meeting were invited to discuss current issues they were facing and to contribute ideas and practical solutions. There was a particularly strong representation from the accounting discipline, perhaps not surprising given the large proportion of international students enrolled in accounting programs. In the early Learning Circle meetings, there was considerable discussion of the literature that emphasises the importance of establishing a social context which is conducive to the development of generic skills (Atkinson et al. 2000; Billing 2007). The significance of promoting intercultural communication skills as a requirement for functioning successfully in the globalised world has been widely acknowledged in the business education literature (Freeman et al. 2009). The issues raised by this literature, especially in relation to the implementation of these ideas in the classroom, were actively debated.

Arising from these discussions, a core group of academics in accounting and construction management decided to develop learning and teaching strategies aimed at promoting intercultural connections among students. The results presented here will focus on curricular changes and outcomes in two undergraduate courses with mostly second year students. The intervention focussed on alliance building between students with different cultural backgrounds, and was implemented in one accounting course and one construction management course in Semester 2, 2011, by two Learning Circle members who closely supported each other in the curriculum development process.

In both courses, academics used Alliance Building activities (from the professional development workshop) in the first tutorial session, to acknowledge the cultural diversity resources in the class, validate the diverse cultural backgrounds of individual students, and encourage participation and sharing in small and large groups. This set the scene for equal status and cooperative multicultural group work right from the beginning. As well, an Academic Skills Program specialist was brought into the classes to talk about working in groups, writing reflective journals, and the time management of group projects.

In the accounting course, students were assigned to work in multicultural groups on a group assignment and in their tutorials. These culturally mixed groups comprising international and domestic students came to be referred to as “rainbow” groups. Students submitted a reflective journal along with their assignments describing the factors that had helped or hindered in communication across cultures in completing their assignments and tutorial work. They were given broad questions on the way their groups had functioned to be addressed in their reflective journals.

Given the narrower range of cultural backgrounds in the construction management course, the classroom activities focussed more on encouraging students to think outside their own experiences and consider alternative world views of particular issues. These students also submitted a reflective journal in which they had been asked to show the development of their ideas about the role of globalisation and cultural diversity in their professional practice.

In the current paper, we focus on evaluation of quantitative student outcomes based on an end-of-semester survey. The qualitative analyses of students’ learning journals are in progress and will not be included here.

## Project evaluation of Canberra student outcomes

### Method

The student participants in the Canberra case study consisted of an intervention group of 140 business undergraduates who were enrolled in courses participating in the IaH Project. Students (n = 59) from two non-IaH undergraduate courses (another accounting course and a tourism course) provided a control group for the evaluation.

The IaH group consisted of 63 males and 77 females, with age ranging from 19 to 48 years (*M* = 24.07, *SD* = 5.14). There were 53 domestic students, 86 international students (or 61.4% of the sample), and one person with unidentified residential status. The non-IaH group consisted of 30 males and 28 females (and one with unidentified gender), with age ranging from 18 to 55 years (*M* = 24.31, *SD* = 6.70). There were 39 domestic students and 20 international students (or 33.9% of the respondents).

### Canberra results on perceived cultural inclusiveness

Table 1 lists the percentage of students agreeing with each of the seven statements on perceived cultural inclusiveness, for the IaH and non-IaH groups separately, and for each of the business schools.

As can be seen from Table 1, in Canberra, IaH business undergraduates consistently reported numerically higher percentages of agreement on each of the statements on perceived cultural inclusiveness, than those among non-IaH undergraduates. Among the IaH students, over two thirds perceived their educational environment to be characterised by teachers encouraging contact between students from different cultural backgrounds, cultural differences being respected in their university, teachers understanding the problems of international students, opportunities in classes for students to learn about different cultures, and classmates being accepting of cultural differences.

We did not find any gender differences in the scaled perceived cultural inclusiveness scores, or any association between age and perceived cultural inclusiveness scores. Hence we ruled out including gender and age as covariates in subsequent analyses testing H1.

As a whole, Canberra business undergraduates enrolled in IaH courses reported statistically more culturally inclusive educational climate (*M* = 3.84, *SD* = 0.54), compared with students in the control group (*M* = 3.52, *SD* = 0.72), *t* (83.99) = 3.05, *p* = .003, Hedges’ *g* = .54. This was a medium effect size.

### Canberra results on cultural learning development

Table 2 lists the percentage of students agreeing with each of the 12 statements on personal cultural learning development, for the IaH and non-IaH groups separately, and for Canberra and Griffith separately.

As can be seen from Table 2, in Canberra, IaH business undergraduates consistently reported numerically higher percentages of agreement on each of the statements on cultural learning development, than those among non-IaH undergraduates. Among the IaH students, over two thirds reported having developed a greater awareness of cultural diversity and a better understanding of cross-cultural interpersonal skills, being more conscious of using cultural knowledge in interacting with people with different cultural backgrounds, being more certain about dealing with adjusting to a new culture, becoming more confident with communicating with people from culturally different backgrounds, becoming more ready to make social contact with culturally different others, and becoming more comfortable participating in multicultural groups.

We did not find any gender differences in the scaled scores of cultural learning development, or any association between age and the cultural learning development scores. Hence we ruled out including gender and age as covariates in subsequent analyses testing H2.

As a whole, Canberra business undergraduates enrolled in IaH courses reported statistically higher levels of cultural learning from participating (*M* = 3.73, *SD* = 0.65), compared with students in the control group (*M* = 3.44, *SD* = 0.75), *t* (197) = 2.92, *p* = .004, Hedges’ *g* = .46. This was a small to medium effect size.

### Lessons shared in Canberra Learning Circles

In Learning Circle meetings, academics who instituted the curriculum changes reported that most of the students’ learning journal entries, especially from international students, reflected positive learning experiences. Students stressed the benefits of working with other students outside their cultural group. But they found the group projects challenging because of difficulties in communication arising from language and cultural barriers and individual personality issues. Regular meetings and patience in dealing with other group members were important. Students who had English as a second language found that asynchronous communication technologies such as email and text messages were a preferable way to communicate as it gave them time to reflect on the content of the communication.

The few negative comments on the experience were from domestic students who felt that the whole theme of internationalisation was being given too much emphasis and that they were already experienced in multicultural communication.

The initial positive outcomes from the intervention have encouraged the academics involved to continue with the IaH interventions in their classes in 2012 and 2013. During this two-year period, these academics have further shared their curriculum innovations and outcomes at BGL- and institution-based teaching and learning workshops and at two (one national and one international) conferences in higher education. The dissemination of these encouraging results has prompted other BGL faculty to adopt some of these teaching strategies and there is now a clear recognition within BGL of the importance of validating students’ diverse cultural backgrounds within the classroom and generally on campus.

### Case 2: Griffith Business School

The Griffith Business School (GBS) has approximately 8,000 students on four campuses in South East Queensland, of whom approximately 3,000 are international students. They come from countries including China, India, Taiwan, Saudi Arabia, and Scandinavia. GBS is an active partner in the Globally Responsible Leadership Initiative, a signatory to the United Nations (UN) Principles for Responsible Management Education and the UN Global Compact. This engagement demonstrates the School’s strong commitment to core values including embracing diversity and demonstrating respect for people of different backgrounds and points of view, and preparing global citizens, with a special focus on the Asia-Pacific Region.

Many GBS academics were cognisant of the highly multicultural nature of the student cohort in their classrooms but were often at a loss about how best to engage students, especially in group work assessment tasks. The two-year IaH Project represented an opportunity to reinvigorate academics’ interest in intercultural learning and teaching pedagogies and to present them with a time-limited initiative in which to engage.

### IaH project processes at Griffith Business School

As described in the Canberra case, GBS lecturers and sessional tutors who attended the professional development workshop were invited to contribute to an ongoing Learning Circle in Intercultural Skills. An informal peer support atmosphere was developed where Learning Circle members critiqued and celebrated their teaching strategies. Initially, the Learning Circle was attended by academics who shared a general interest in the topic but were not involved directly in implementing curricular changes. Owing to workload and time pressure, their attendance waned over time, leaving only those academics who had elected to participate in the design, delivery, and evaluation of IaH interventions. Formal Learning Circle meetings were held at least once every semester, over an 18-month period. Informal communication about the project occurred between members and the faculty leader on an almost weekly basis in the semester when the curriculum changes were first introduced.

Discussions in the Learning Circle generated particular interest from academics teaching into a compulsory first year management course taught each semester across three campuses of the university. The primary focus of weekly tutorials in this course was participating in and leading problem-based group discussions. Academics found these tasks to be very challenging for introverted students and students from culturally and linguistically diverse backgrounds. Interaction between domestic and international students was a particularly challenging aspect of tutorials for both tutors and students. This was exacerbated because 25% of course assessment was based on students’ ability to participate and lead case analysis and discussion in small groups of three to four students who met weekly in tutorials.

On hearing about Canberra BGL’s success in using EXCELL Alliance Building in multicultural group work linked to assessments, GBS Learning Circle members became interested in exploring how the use of both Alliance Building and Cultural Mapping tools might benefit their first year management students in participating in and leading problem-based group discussion. During GBS Learning Circle meetings, it became evident that academics and particularly sessional tutors welcomed the opportunity for further professional development in the use of these tools. The one-day workshop program did not allow sufficient time for the academics to fully understand the tools, experiment with them, and reflect on how they could be incorporated.

As a result, an additional three-hour professional development workshop on the use of the EXCELL tools was offered to sessional tutors. The workshop focused on how Alliance Building activities could be used in the first tutorial of the semester as part of “Getting to Know You”, and how the Cultural Mapping method could be integrated in Weeks three and four of the semester (related to the group work assessment task). Cultural Mapping could provide a structured, modelled approach to teaching group participation and leading skills to students, especially international students from culturally and linguistically diverse backgrounds.

The Griffith student outcomes presented here will focus on those in the first year management course in Semester 1, 2012. Based on lessons from evaluating Canberra student outcomes, we attempted to minimise confounding from different year levels by recruiting our control group from another large first year course at Griffith University. This way, we also hoped to be able to recruit a sufficiently large comparison group, to allow for sub-group analyses for domestic and international students separately.

## Project evaluation of Griffith student outcomes

### Method

Students from two Griffith courses – an introductory GBS course (in management) that was part of the IaH Project (*n* = 211) and a non-IaH introductory course in tourism (*n* = 84) - responded to a survey about their student experience.

The IaH sample comprised 91 males and 118 females, aged from 17 to 64 years (*M* = 23.70, *SD* = 6.89). There were 177 domestic students and 34 international students (or 17% of the respondents).

The non-IaH sample comprised 29 male and 52 female respondents, aged from 17 to 44 years (*M* = 21.14, *SD* = 4.33). There were 48 domestic students and 36 international students (or 60.2% of the respondents).

Owing to the differing percentages of international students in the IaH and non-IaH groups in this case study, and the sufficiently large sub-sample size, we conducted between-groups analyses for the sample as a whole, as well as for domestic and international students separately.

### Griffith results on perceived cultural inclusiveness

As can be seen from Table 1, Griffith IaH first year undergraduates consistently reported numerically higher percentages of agreement on each of the statements on perceived cultural inclusiveness, than those first year students from the control group. Among the IaH students, over two thirds perceived their educational environment to be characterized by teachers encouraging contact between students from different cultural backgrounds, cultural differences being respected in their university, and classmates being accepting of cultural differences.

As a whole, at Griffith University, first year students (*n* = 211) in the IaH course reported statistically higher levels of cultural inclusiveness from participating in the course (*M* = 2.17, *SD* = 0.82), compared with students (*n* = 83) enrolled in the control group (*M* = 2.64, *SD* = 0.68), *t* (292) = -4.59, *p* < .001, Hedges’ *g = -*0.60. This represented a medium to large effect size.

In sub-group analysis, domestic Griffith University students in the IaH course (*n* = 177) reported statistically higher levels of cultural inclusiveness (*M* = 2.15, *SD* = 0.82), compared with domestic students (*n* = 47) in the control group (*M* = 2.42, *SD* = 0.57), *t* (103.35) = -2.63, *p =* .01, Hedges’ *g* = -0.35. This represented a small to medium effect size.

International students enrolled in the IaH course (*n* = 34) reported statistically higher levels of cultural inclusiveness (*M* = 2.29, *SD* = 0.80), than did international students (*n* =36) in the control group (*M* = 2.92, *SD* = .73), *t* (68) = -3.477, *p <* .01, Hedges’ *g* = -0.84. This was a large effect size.

### Griffith results on cultural learning development

As can be seen from Table 2, first year Griffith IaH students consistently reported numerically higher percentages of agreement on each of the statements on personal cultural learning development, than those among non-IaH first year students. Among the IaH students, over two thirds reported having gained greater awareness of the role of culture in their chosen field of study, being more conscious of using cultural knowledge in interacting with people with different cultural backgrounds, being better prepared to adjust their cultural knowledge as they interact with people from an unfamiliar culture, and becoming more comfortable participating in multicultural groups.

As a whole, Griffith IaH students (*n* = 210) reported higher levels of cultural learning from participating in the course (*M* = 2.27, *SD* = 0.76), compared with students (*n* = 84) in the control group (*M* = 2.44, *SD* = 0.66), *t* (292) = -1.81, Hedges’ *g = -0.23*. This represented a small effect size, and the *t* test result was statistically significant only when a one-tailed test was used, *p* = .036.

Sub-group analysis revealed that domestic Griffith students enrolled in the IaH course (*n* = 176) did not report statistically higher levels of cultural learning from participating in the course (*M* = 2.28, *SD* = 0.78), compared with domestic students (*n* = 48) in the control group (*M* = 2.33, *SD* = 0.71), *t* (222) = -0.36, Hedges’ *g = -*0.07. This suggests a statistically insignificant effect.

In contrast, Griffith international students in the IaH course (*n* = 34) reported statistically higher levels of cultural learning from participating in classes (*M* = 2.17, *SD* = 0.66), than did international students (*n* = 36) in the control group (*M* = 2.58, *SD* =0.57), *t* (68) = -2.80, *p* = .008, *g = -0.68*. This represented a medium to large effect size.

### Lessons shared in Griffith Learning Circles

Learning Circle members shared their reflections about how the use of an Alliance Building activity around the cultural origins of every student’s name has turned what might otherwise be a routine “Getting to Know You” exercise, into an enjoyable, surprisingly instructive way to learn about cultures and cultural differences. The abstract concept of culture becomes far more real and personal, and cross-cultural comparisons become possible. Academics reported that even those tutors who were not able to attend either of the professional development opportunities were able to learn to debrief the exercise quite easily.

Academics reflected on the Cultural Mapping tool as a very useful tool to teach group participation and group leadership skills. The written step-by-step maps acted as visual models to help first year students remember appropriate verbal and non-verbal communication strategies. Informally and in end-of-course evaluations, students reported they had a positive experience in tutorials and many stated they experienced more cooperative peer interactions when involved in small group class discussions.

After learning from academics involved in the IaH course, two other members of the Learning Circle decided to use the Alliance Building and Cultural Mapping tools in a third year intercultural management course in two modalities – on-campus and online - in subsequent teaching periods. Alliance Building activities were built into the first tutorial (on-campus) and in the first online module. Students were taught to apply the Cultural Mapping tool in intercultural case studies where communication breakdown was evident. For example, maps were developed to address how to give feedback to an employee whose behaviour was culturally inappropriate. Adaptation of the tools and evaluation of their impact on students’ learning in this third year course is the focus of ongoing analysis.

## Discussion and evaluation

In both case studies, faculty reported considerable challenges in teaching multicultural classes and engaging domestic and international students in group work. In line with previous research (Woods et al. 2011), these challenges included issues around English competence and reticence about collaborating in assessment groups. However, Learning Circle members have observed that collaborating with colleagues to implement strategic, structured active learning interventions such as those based on EXCELL Alliance Building and Cultural Mapping tools, could bring about more productive social interactions in group discussion and completing group assignments in culturally mixed classes.

### Positive student outcomes especially for international students

For each of the case studies, results from student surveys showed that students who had completed courses that were part of the IaH project intervention reported significantly greater overall perceived cultural inclusiveness in multicultural classes, compared with students in the control group. Students in the IaH intervention group reported significantly greater levels of cultural learning development than students in the non-intervention courses. Overall, we found support for both of our hypotheses regarding quantitative student outcomes. Considering also the experiences and observations of Learning Circle faculty members, it would appear that IaH-type interventions involving professional development and continual support for faculty are important for promoting improved student interactions and engagement in learning tasks in culturally mixed classes via a more culturally inclusive educational environment and opportunities for personal cultural learning development.

Subgroup analyses in the Griffith case study indicated that both the international and domestic students in the intervention group reported greater levels of perceived cultural inclusiveness in their educational environment, than their counterparts in the control group. However, in terms of cultural learning development among students in culturally mixed classes, we found a statistically significant difference between IaH and non-IaH international students (with a medium to large effect size), but no difference between IaH and non-IaH domestic students.

This differential outcome for Griffith students has two important implications that will require future research. First, international students could especially benefit from educational interventions designed to enhance intercultural competence development, perhaps because, as cultural newcomers mainly from non-English-speaking backgrounds, their prior confidence and skills in communicating across cultural and linguistic divides are lower than those among domestic students. Canberra Learning Circle members have also found international students’ learning journals reflecting particularly favourable comments of their cooperative work in culturally mixed groups.

Second, the much higher proportion of international students in the Griffith control group might have facilitated domestic students’ cultural awareness and intercultural skills out of mere exposure (as in the intergroup contact literature, see Pettigrew and Tropp 2006) or sheer necessity, or both. Further research attention is needed to gain a better understanding of the experiences of domestic students, especially in relation to the nature of their interactions in multicultural group work.

We further note various methodological limitations in the evaluation of student outcomes in our case studies. The present quasi-experimental design made use of real-life courses; we cannot rule out that the control condition courses such as the tourism courses could also contain elements of intercultural learning in their curriculum. Future investigations could minimise potential confounding by comparing pre- and post-intervention student cohorts from the same course, and by using a pre- and post-intervention design within the same cohort. Additional indicators of students’ educational outcomes (such as engagement in learning and actual academic performance) and intercultural contact outcomes (such as intercultural friendships), could also be used. Further research could also use qualitative methods, including content analyses of reflective journals on cultural learning and focus groups with international and domestic students.

### Lessons from professional development and learning circles

The IaH project used a cross-institutional, cross-disciplinary community of practice approach to follow up on a one-day professional development workshop and to support academics in making curricular interventions that promoted an inclusive educational climate and facilitated intercultural learning. Both case studies have shown that while it was resource intensive to continually mentor academics engaged in the project, the Learning Circle approach enabled supportive peer-to-peer learning for academics committed to enhancing intercultural interactions in highly culturally diverse student cohorts.

However, faculty leaders at both business schools have noted the challenge of attracting and retaining Learning Circle members on “teaching-research” track, who were torn between wanting to improve teaching and learning practices, and at the same time needing to be research productive to maintain their academic appointments. The leaders were able to partly address this issue by encouraging and mentoring small groups of Learning Circle members to plan and conduct research around educational interventions, and to present their work at conferences and via collaborative research publications.

Another challenge was staff turnover and the large numbers of sessional tutors involved in teaching compulsory undergraduate courses. This suggests that considerable attention needs to be given to continual professional development of tutors. Given that tutorials were where students were taught Alliance Building and Cultural Mapping, tutors need to be taught the tools intensively and opportunities need to be provided for tutors to reflect on their use and to discuss where modifications need to be made so that learning and teaching is optimal.

### Institutional support in embedding intercultural competence development

The results reported here came from early systematic attempts to implement strategies to promote intercultural learning in the business classrooms of two Australian universities. A major challenge of implementing the teaching innovations has been engaging and sustaining faculty throughout the project and beyond. We have identified a need for providing an ongoing professional development opportunity, such as through intercultural training and learning circle support, in building intercultural capability in academics at all levels of appointment. This opportunity needs to model how curricular changes can be made and include practical support for faculty in making the changes. Discipline-based learning circles can provide a genuine opportunity for embedding intercultural competence development at various parts of a program of study.

Importantly, institutional requirements (of increasing students’ intercultural capability via course content, teaching methods, and assessment) in program review and re-accreditation, could ensure that relevant curriculum changes are made and reviewed periodically. Cross-disciplinary sharing of intercultural learning innovations, via institutional as well as school-based fora and web resources, will further enhance the exchange of good practices vital for preparing graduates for culturally diverse and global workplaces.

## Conclusions

Business faculty reflections on the benefits of the Internationalisation at Home Project concur with survey results that support the hypotheses about positive student outcomes in terms of perceived cultural inclusiveness and cultural learning development. Moreover, the same patterns of findings have been identified in two case studies involving different disciplines in two Australian universities, and where faculty have adapted their learnings from the professional development and learning circles, in ways that suited their subject matter and personal teaching styles.

Overall, the IaH project implementation in multicultural group work at two business schools has resonated with previous research findings that the development of students’ intercultural capability does not happen automatically in culturally mixed classes. Improvement in students’ intercultural competence – whether they are domestic or international students – requires planned interventions through curriculum changes involving the embedding of intercultural competence development. The existing cultural diversity in the business student population affords real-life classroom opportunity for implementing active, experiential learning strategies vital for the development of cultural competence among both home and especially international students.
